# A Comparative Analysis of Deep Learning Convolutional Neural Network Architectures for Fault Diagnosis of Broken Rotor Bars in Induction Motors

**DOI:** 10.3390/s23198196

**Published:** 2023-09-30

**Authors:** Kevin Barrera-Llanga, Jordi Burriel-Valencia, Ángel Sapena-Bañó, Javier Martínez-Román

**Affiliations:** Institute for Energy Engineering, Universitat Politècnica de València, Camino de Vera s/n, 46022 Valencia, Spain; kebarlla@upv.edu.es (K.B.-L.); jorburva@die.upv.es (J.B.-V.); asapena@die.upv.es (Á.S.-B.)

**Keywords:** artificial intelligence, convolutional neural network, deep learning, induction machine, broken bar detection, automatic diagnosis

## Abstract

Induction machines (IMs) play a critical role in various industrial processes but are susceptible to degenerative failures, such as broken rotor bars. Effective diagnostic techniques are essential in addressing these issues. In this study, we propose the utilization of convolutional neural networks (CNNs) for detection of broken rotor bars. To accomplish this, we generated a dataset comprising current samples versus angular position using finite element method magnetics (FEMM) software for a squirrel-cage rotor with 28 bars, including scenarios with 0 to 6 broken bars at every possible relative position. The dataset consists of a total of 16,050 samples per motor. We evaluated the performance of six different CNN architectures, namely Inception V4, NasNETMobile, ResNET152, SeNET154, VGG16, and VGG19. Our automatic classification system demonstrated an impressive 99% accuracy in detecting broken rotor bars, with VGG19 performing exceptionally well. Specifically, VGG19 exhibited high accuracy, precision, recall, and F1-Score, with values approaching 0.994 and 0.998. Notably, VGG19 exhibited crucial activations in its feature maps, particularly after domain-specific training, highlighting its effectiveness in fault detection. Comparing CNN architectures assists in selecting the most suitable one for this application based on processing time, effectiveness, and training losses. This research suggests that deep learning can detect broken bars in induction machines with accuracy comparable to that of traditional methods by analyzing current signals using CNNs.

## 1. Introduction

Induction machines (IMs) play a crucial role in modern industrial processes, powering various machinery and equipment. The diagnosis of faults in IMs has become an essential aspect of condition-based maintenance (CBM) programs. Unexpected breakdowns of IMs can lead to significant economic losses due to production downtime. To address these challenges, fault diagnosis techniques based on motor current signature analysis (MCSA) have gained popularity due to their simplicity, low implementation requirements, and ability to detect multiple faults simultaneously [1,2,3,4,5,6]. MCSA is commonly used for detection of broken bars in IMs. Extensive studies have applied signal processing techniques like wavelet transforms, Wigner–Ville distributions, Hilbert transforms, and Prony analysis to extract fault signatures from stator currents [7,8]. Reviews offer overviews of MCSA-based broken bar detection using time and frequency domain analysis of stator currents [8,9].

Despite its advantages, the industrial application of MCSA in hostile environments and real working conditions remains challenging. The identification of fault indicators in the form of spectral lines in the current spectrum can be difficult due to the presence of numerous lines generated by electromagnetic interference. This problem is further aggravated for incipient faults, where fault signatures have very low amplitudes, and under low-slip conditions, where the leakage of fundamental current masks fault harmonics occurring at proximal frequencies.

Over the years, advancements in technology have led to the development of various approaches to address these challenges, and the field has delved deeper into techniques specifically tailored for the detection of broken bar faults in induction motors. In the context of vibration signals, the authors of [10] developed an enhanced cyclic modulation spectral analysis approach using continuous wavelet transform to extract fault signatures with high accuracy. This method was shown to be effective in diagnosing one broken rotor bar under different load conditions. The research reported in [11] proposed combining Hilbert transform of stator current spectra with artificial neural networks for broken bar fault diagnosis in indirect field-oriented control-fed motors. Their technique quantified fault severity based on sideband amplitudes. The research reported in [12] provided a comprehensive review of recent broken bar detection methods for line-fed and inverter-fed induction motors published after 2015. Their analysis highlighted key features and provided comparisons of the reported techniques. In [13], the authors introduced an inverse thresholding approach applied to startup current spectrograms to enhance fault feature visibility for broken rotor bar detection. Their method weakened fundamental components to make fault frequencies more apparent.

Model-based techniques have been explored for motor fault diagnosis and prognosis. The authors of [14] presented an integrated design method combining active fault diagnosis and tracking control to detect incipient faults while maintaining normal closed-loop operation. The authors of [15] proposed utilizing zonotopic observers and MANFIS models for robust fault diagnosis. In [16], it was demonstrated that a failure due to breakage of two bars located at polar passage in the air gap can mask the failure harmonic and make its detection by spectral techniques unfeasible. These studies demonstrate active research interest and progress in applying current signature analysis for broken bar fault diagnosis in induction motors.

Recently, the use of expert systems has gained prominence as a means of enhancing fault detection accuracy, especially in situations with limited or unrepresentative fault data. These systems facilitate improved reliability by leveraging accumulated domain knowledge to identify fault signatures [17], thus allowing for the efficient extraction of fault characteristics and patterns [18] and enabling timely corrective actions to minimize downtime [19].

Deep learning, a subset of machine learning, has emerged as a tool for induction machine fault classification, addressing the scarcity of fault data. Notably, methods such as the use of neural networks, autoencoders, and LSTMs have shown promising results when applied to raw sensor data [20,21]. Transfer learning approaches overcome data limitations [22,23]. Vibration analysis with classifiers like KNN has also been explored [24]. Advancements incorporate intelligent diagnostics [25] and real-time neural classifiers [26]. Hybrid statistical and machine learning approaches achieve high accuracy [23]. Transfer learning and vibration imaging help to understand the spectral behavior of faults [27] and vibration-based damage detection methods using dictionary learning [28].

Of particular interest in recent developments is the application of convolutional neural networks (CNNs) in crafting diagnostic solutions based on time-series signals and images. These networks excel at capturing localized spatial relationships in signals and images through convolutions, thereby adeptly learning multiscale hierarchical features via deep architectures [20]. This growing interest is epitomized in studies proposing methods based on branch current analysis [29] for detection of surface damage with convolutional neural networks [30] and damage image classification [31], as well as deep neural network methods for extraction of damage features [32] and sensor fusion [33].

Motivated by these advancements, this work proposes a novel CNN-based method for automatic detection and quantification of broken rotor bars in induction machines using images of current signals. The main objectives and contributions are summarized as follows.

1. An approach is proposed that utilizes convolutional neural networks (CNNs) to accurately detect the number of broken bars in induction machines based on images of current absorbed by a single-fed stator phase versus the blocked-rotor angular position.
2. A time-series image preprocessing technique is introduced that enhances the learning capabilities of CNN models.
3. A comparative analysis of different CNN architectures for the fault detection system is performed, taking into consideration factors such as processing time, classification efficiency, and training losses.
4. A novel visual interpretation of the filter activations in the feature maps of the selected CNN architecture before and after training is conducted, providing insights into how the model interprets and learns patterns from the data. This represents an important novelty of this work, as it aids in understanding the process that the CNN undergoes during its training process, an issue seldom addressed in the technical literature.

Our preliminary experiments explored logistic regression and support vector machines (SVM), but these methods encountered limitations in handling the nonlinear relationships and variations present in the dataset [34,35]. Consequently, we transitioned to exploring artificial neural networks (ANNs), which demanded a substantial and diverse dataset to capture relevant features and learning patterns optimally [36]. Addressing this, the dataset was transformed into two-dimensional images, enhancing the model’s ability to perceive visual and spatial features associated with faults in the current versus angular position signals. The use of images as input representation circumvents the need for hand-crafted feature extraction from raw signals. The models can automatically learn relevant visual patterns and spatial relationships within current images to accurately classify fault severity.

The proposed technique aims to leverage the capabilities of CNN models to accurately identify the number of broken bars in induction machines. A comparative evaluation of various CNN architectures is conducted to ascertain the most suitable model for this problem, accompanied by a novel visual analysis of feature maps, offering profound insights into the interpretation of current signature images. Table 1 summarizes the proposed approach, key contributions, and limitations.

The remainder of this paper is organized as follows. Section 2 describes the proposed method, the dataset, and the CNN training process. Section 3 presents the comparative results and analysis of model feature maps. Section 4 provides a discussion of the results. Finally, conclusions are presented in Section 5.

## 2. Methodology

### 2.1. Overview

For several years, research has been actively conducted on the development of fault detection systems in electrical machines. A model of induction machines with spatial harmonics was designed to detect faults using the convolution theorem [1]. Additionally, short fourier transform (SFT) has been utilized to diagnose faults in transient induction machines [2]. Recent explorations include the implementation of artificial intelligence in the diagnosis of electrical machines, such as the development of an automatic fault diagnosis system for transient induction motors using expert systems [3]. The proposed workflow involves analyzing and modeling potential faults in induction machines, simulating these faults using electromagnetic simulation software, and generating datasets that represent different fault scenarios. From these datasets, fault detection and diagnosis algorithms and systems are developed. Tests and experiments are then conducted on a test bench, subjecting the induction motors to controlled fault situations to evaluate the effectiveness of the detection and diagnosis systems. Lastly, the results obtained from the data collected on the test bench are analyzed and compared with the expected outcomes, and improvements are made based on these analyses.

**Figure 1:**
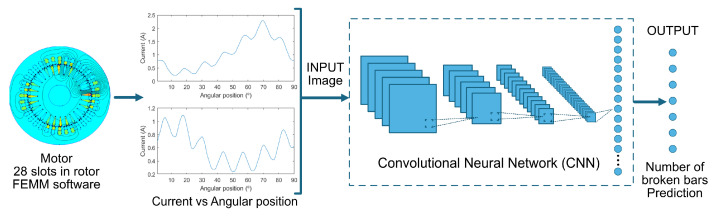
General structure of the CNN-based system for automatic broken bar detection in rotors using FEMM software data. Data are converted into images representing current vs. angular position for different broken bar scenarios. The system evaluates multiple architectures and predicts the number of broken bars.

The objective of this work is to develop, train, compare, and validate an automatic system for the detection and quantification of the broken bar fault in the rotor of an induction machine. This is achieved using the phase current when a single stator phase is fed as a function of the rotor angular position. The general scheme of the system is depicted in Figure 1.

The input comprises images of the signals representing current vs. angular position for every combination of the number of broken bars and their possible relative positions in the rotor cage. As the output, the system produces seven possible predictions based on the number of broken bars, irrespective of their spatial location (whether consecutive or not):

- Healthy motor;
- One broken bar;
- Two broken bars;
- Three broken bars;
- Four broken bars;
- Five broken bars;
- Six broken bars.

The images undergo preprocessing as described in the following section. The system uses a deep learning model (CNN) to classify the images automatically. The model is composed of convolutional filters that scan the images and extract the highlighted features from each filter. The image is reduced in size and increased in depth until it becomes a feature vector. Subsequently, fully connected layers are used to obtain a probabilistic vector with dimensions of 1×7, where 7 corresponds to the systems prediction outputs. The result is provided based on the position with the highest prediction value. Once the system is trained, the performance of various architectures is compared through conducting quantitative evaluation with classification metrics. Finally, the learning of the selected model is analyzed, and the feature maps before and after training of the CNN are visualized with the aim of finding the convolutional layer of the network that learns the most and achieves the best performance.

### 2.2. Image Dataset

The finite element method magnetics (FEMM) software [37] was used to simulate a commercial three-phase, 28-bar induction motor. The motor specifications for the simulation are as follows: a power rating of 1.1 kW and a frequency of 50 Hz, operating at a voltage of 230/400 V with a current of 2.7/4.6 A and achieving a speed of 1410 r/min with a power factor of 0.8. The motor’s structural characteristics are detailed as follows: an effective magnetic core length of 120 mm, radius at the midpoint of the air gap of 54.11 mm, and an air gap length of 0.28 mm. The stator is constructed with a three-phase winding scheme housed in 36 slots, each accommodating 27 wires, and exhibits a winding pitch of 7/9. Additional features include a slot-opening width of 2.1 mm, a phase resistance of 7.68 W, and an end winding leakage of 2.3 mH. On the other hand, the rotor adopts a squirrel-cage winding design, comprising 28 bars with a slot-opening width of 1.4 mm and a skew equivalent to one slot pitch. The rotor bars exhibit a resistance of 0.00202 mW and an end winding leakage of 2.45 × $10^{-5}$ mH.

In the simulation, signals of phase current against the rotor’s angular position were derived by supplying AC voltage to a single stator phase, thereby ensuring that the rotor remained stationary at every specific angular position. Essential images were obtained to establish a dataset for neural network training. Table 2 enumerates the number of images used for training, validation, and testing of the models. The Samples column indicates the data collected for each number of broken bars.

Given that the initial angular position can vary depending on the angular reference point and the position of the broken bars, we proposed generating new images representing different combinations that can occur when a bar breaks [38]. Adjusting the initial angular position by shifting it from 0 to 90 degrees in 1-degree intervals is suggested. This method creates a rotation effect in the image series, capturing the variations in current behavior throughout the complete angular range. By shifting the initial angle, a series of images representing the changes in current as the angular position progresses is produced, resulting in a comprehensive dataset. The total number of these combinations is specified in the Combinations column of Table 2. This process allows the network to recognize the patterns and dynamics of current behavior in response to different angular positions, enhancing its ability to detect and classify broken bars in the induction motor.

For the proper training of the convolutional neural network, the dataset was divided into three groups: training, validation, and testing [39]. The network learns the variation in current as a function of the number of broken bars using the training and validation sets, and the testing set serves as a proof of concept to assess the accuracy of the detection corresponding to the network capability. To partition the database, the Pareto principle of 80/20 was applied [40]. This principle suggests that 20% of the data represents the main characteristics of the majority group of 80%. While the 80/20 principle served as a fundamental guide, the optimal partition ratio was established through rigorous testing of multiple partitions. Specifically, the CNN models were trained and tested using 60/40, 70/30, 80/20, and 90/10 ratios. Classification performance was evaluated using classification metrics. Among the different ratios tested, the 80/20 split consistently tended to produce the best results in terms of predictive accuracy. The analysis of the metrics obtained from the 80/20 partition configuration is detailed in Section 3. Building upon this foundation, 80% of the database was allocated for training, and the remaining 20% was used for testing. the training set was further split into training and validation sets to evaluate the effectiveness of the CNN classification. The allocation of these datasets is indicated in the Train, Val, and Test columns of Table 2.

**Figure 2:**
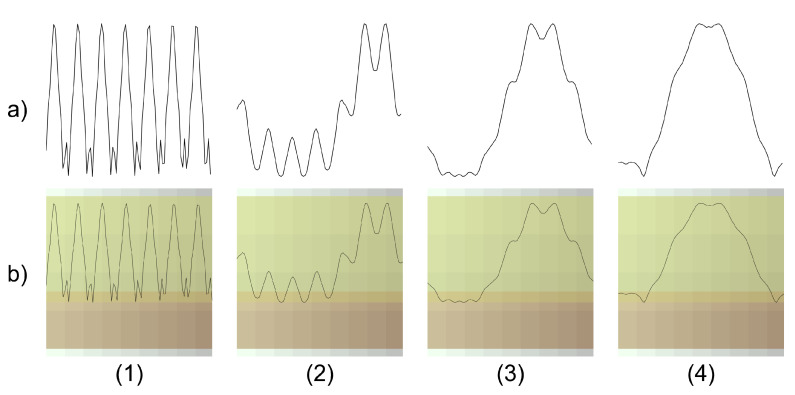
Preprocessingtechnique varying the background in gradient colors. (**a**) Examples of current signal vs. angular position in a healthy motor (**1**) and in motors with two (**2**), four broken (**3**), and six broken bars (**4**). (**b**) Examples of current signal vs. angular position in images with a degraded background of a healthy motor (**1**) and motors with two (**2**), four (**3**), and six broken bars (**4**).

In the current study, a challenge was encountered in achieving satisfactory accuracy during the training of the CNN. Through training tests, it was observed that the feature maps of the trained models were heavily influenced by the soft background of the time-series images, leading to erroneous training results. To address this issue, a novel image preprocessing technique was introduced, which involved the modification of the current vs. angular position signals in the background of the images. Specifically, after conducting multiple experiments with various backgrounds while training the networks, it was discerned that a grid with gradient colors yielded the best outcomes. As illustrated in Figure 2, the vertical component of this gradient is determined by the degree of the angular position, and the horizontal component influenced by the normalized values of the current. It was decided to detail this particular procedure in this paper due to its superior performance. It is worth noting that, according to extensive testing, this technique demonstrated notably promising results, significantly enhancing the classification capabilities of the CNN. A more detailed evaluation of how this preprocessing technique impacts model learning can be found in Section 3.2.

In addition to the image preprocessing technique, data balancing and augmentation were crucial in enhancing the performance of the CNN [41,42]. The objective of data balancing is to ensure roughly equal sample sizes for each class, eliminating potential biases. In some instances, balancing entailed augmenting under-represented classes, while in others, it necessitated reducing over-represented classes. Testing revealed optimal performance when the dataset was balanced to approximately 3000 samples per class for both training and validation.

To achieve this balance using data augmentation techniques, we aimed to introduce variations in the image perspective while preserving its essential content. Specifically, techniques such as rotating the images at various angles of inclination and vertically flipping them were utilized. While the original signal is inherently one-dimensional and does not naturally exist in a 2D rotated state, these rotations are not intended to mimic a potential real-world input. Instead, they aim to diversify the understanding of the model of signal attributes. With the addition of color gradients in the background, rotations allow for a more intricate encoding of signals based on these gradients. By inverting the signal, the vertical flip retains its essential features in a reversed order, maintaining validity. Randomly applying these augmentations prevented overfitting and unintended biases. The Data Augmentation column in Table 2 details the sample counts for each class.

### 2.3. Proposed Training

During the preliminary research stage, an expansive analysis encompassing CNN architectures ranging from AlexNet to EfficientNetB7 was undertaken. The architectures were evaluated based on the metrics elucidated in the results section. To foster consistency in comparisons, the same training methodology outlined in this section was followed. We propose working with the same initial parameters, including the number of training epochs. This approach facilitated the identification of a subset of architectures demonstrating high performance on the dataset described in Table 2. The architectures were investigated:

- Inception V4: This architecture utilizes filters of different sizes to capture various scales of features in the dataset, enabling effective identification of different characteristics of the broken bars. The outputs from these filters are concatenated and passed to the next layer [43].
- NasNETMobile: Designed for lightweight training, NasNETMobile employs larger convolution filters to reduce computational complexity while achieving notable performance. The architecture was pretrained on a sizable dataset, facilitating the extraction of high-level features relevant to the classification task [44].
- ResNET152: The ResNET152 architecture incorporates skip connections, enabling direct connections between layers during training. This feature ensures efficient information flow and addresses the vanishing gradients challenge. Given its depth and capability of capturing intricate features, ResNET152 proves suitable for the classification problem at hand [45].
- SeNET154: SeNET154 is a squeeze-and-excitation network that adaptively calibrates the response of each class by modeling interdependencies between layers. It utilizes compression and excitation techniques to enhance the representation capacity of the network [46].
- VGG16: VGG16 employs multiple 3×3 convolutional kernels to capture hierarchical representations of the dataset. With its 13 convolutional layers and 3 dense layers, VGG16 offers relatively fast processing while maintaining good performance [47].
- VGG19: As an extended version of VGG16, VGG19 includes additional convolutional layers to capture more complex patterns and features. Although it requires more processing power in the training process, VGG19 can achieve classification results comparable to those of more complex architectures [48].

The learning of the network happens in the convolution process [49], in which a filter (kernel) is applied to the input image to derive a feature map representing the main characteristics. Mathematically, this can be represented by Equation (1), where the output feature map ($M_{m,n}$) is obtained by summing the element-wise product of the kernel (*k*) and the input image (*x*) over all possible positions (i,j).

(1) $$M_{m,n}=\sum_{i}\sum_{j}k[i,j]x[m-i,n-j]$$

where *m* and *n* represent the rows and columns of the feature map, respectively.

In neural network training, the pivotal role of input images and their corresponding output categories becomes evident. Upon processing these images, the network formulates predictions rooted in previously identified patterns. Each cycle of this process, where the dataset navigates forward and backward through the network, is termed an epoch. During these epochs, the network’s primary objective is to refine its weights and biases [50], continuously minimizing the gap between actual and predicted categories. This refining process leverages the backpropagation technique, which evaluates the network’s outputs against desired results, adjusting weights according to identified discrepancies. A neural network essentially functions by aggregating all its inputs, modulated by specific weights and offset by biases or learning errors.

Building upon this fundamental understanding, in the current study, we prepared the input images by transposing 1D current wave forms against the angular position rendered on a 2D grid background, generating an image dimension of 320×180 pixels. For compatibility with the chosen architectures, these images were resized to a resolution of 224×224 pixels—a size that retains pertinent waveform characteristics and aligns with standardized values prevalent in the analyzed architectures. As these processed images are fed into the model input layer, the subsequent intermediate layers retain the unique configurations of their respective architectures. The culmination is an output layer with seven nodes corresponding to the seven classes of potential: healthy motor and motors with one to six broken bars. This categorization allows the model to quantify the number of broken bars based on the input signal.

Given the computational intensity of this process, optimization becomes indispensable. The Adaptive Estimation of Moments (Adam) optimizer [51] plays a crucial role in error reduction, computing the mean gradient over epochs to alleviate the computational strain of training, as depicted in Equation (2). With each epoch, the network evolves, and through the accumulation of these epochs, the CNN’s capability of identifying image patterns and features intensifies [52].

(2) $$\begin{array}{cc} m= & \beta_{1}\;\cdot \;m+\left(1-\beta_{1}\right)\;\cdot \;\Delta W\\ v= & \beta_{2}\;\cdot \;v+\left(1-\beta_{2}\right)\;\cdot \;\Delta W^{2}\\ W= & W-\frac{\alpha \;\cdot \;m}{\sqrt{v}+\varepsilon } \end{array}$$

where *m* and *v* represent the Adam moments (the mean of the gradients over time); *W* is the training weight; $\beta_{1}$ and $\beta_{2}$ are the variance parameters, set at 0.9 and 0.99, respectively; and α is the learning rate at 0.001 for the backward and forward processes.

Based on this optimization approach, the selection of an appropriate error metric congruous with the essence of the task emerges as another cornerstone of the training process. For the multiclass classification problem addressed in this research, the cross-entropy loss [53] constitutes an optimal choice. Fundamentally, this loss function quantifies the divergence between the predicted class probability distribution and the true label distribution. This makes it an apt metric to evaluate the alignment of the model inferences with the ground truth labels. The cross-entropy loss is defined as:

(3) $$L\left(y,p\right)=-\sum_{c=1}^{C}y_{c}\log\left(p_{c}\right)$$

where L(y,p) denotes the cross-entropy loss between the true labels (*y*) and predicted probabilities (*p*). The index *c* iterates over the number of classes (*C*), and $y_{c}$ represents the binary indicator for the true class label (*c*), while $p_{c}$ corresponds to the predicted probability for class *c*. By minimizing the cross-entropy between predictions and ground truth, the model is optimized to align inferences with true labels. This enables efficient multiclass learning. In conjunction with the aforementioned techniques, optimizing other salient hyperparameters is imperative for efficacious training. By harnessing the capabilities of the PyTorch library, iterative testing enabled fine-tuning of these parameters. A batch size of eight was selected based on memory limitations and model convergence criteria. The Adam optimizer proved instrumental in engendering optimal and stable training. However, prudent tuning of the learning rate was requisite. An initial value of 0.001 was chosen, followed by gradual decay using a one-cycle policy to adroitly maneuver the model through potential saddle points. To mitigate overfitting, L2 regularization was integrated into the training protocol via weight decay. Additionally, data augmentation parameters achieved a balance between removing biases and retaining integral signal characteristics.

For the purpose of this study, a fixed count of 20 training epochs was identified as optimal based on a series of considerations. Predominantly, the training losses tend to reach a stable and minimal threshold around this mark. Restricting training to these 20 epochs also streamlines the algorithm, curtailing computational demands and curbing the risk of overfitting. Furthermore, this limitation offers a safeguard against the model descending into local minima, which could impede optimal performance. While an approach centered on achieving the least error may seem appealing, a predefined epoch count ensures consistency and a more coherent comparison between models.

**Figure 3:**
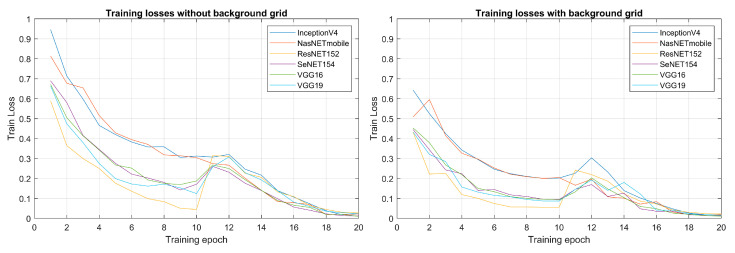
Comparison of training losses in 20 training epochs for the electrical current in relation to the angular position images. The figure is divided into two parts: the first part shows the training losses before the addition of the background grid in image preprocessing, while the second part shows the training losses after the addition of the background grid.

During the learning process, a reduction in training losses was observed for the current in relation to the angular position images across the six architectures, as depicted in Figure 3. The figure comprises two graphs; the first compares training losses prior to incorporating the background grid in the image preprocessing, while the second compares training losses after the inclusion of the background grid during preprocessing.

**Figure 4:**
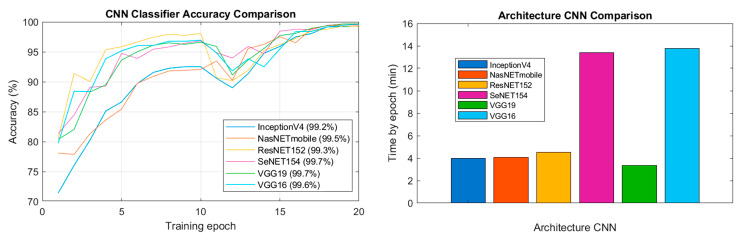
Comparison of training accuracy and processing time per epoch for CNN architectures over 20 training epochs.

To assess the effectiveness of training across all architectures, the closeness of the prediction to the specific label value of each broken bar must be calculated. Accordingly, accuracy during training and in the proof of concept is measured. Accuracy [54] is a statistical measure commonly employed in binary or multiclass classifications and is applicable in this context. This measure evaluates the correct predictions (true positives and true negatives) out of the total cases examined by estimating the probability before and after the input of images into the CNN [55]. The training accuracy over time for each of the architectures described in this research was compared to detect the number of broken bars in a 28-bar induction motor rotor. Figure 4 illustrates the evolution of accuracy, as well as the average processing time for each training epoch.

## 3. Evaluation of the Models

The purpose of this section is to assess the effectiveness of the models in detecting broken bars in induction motors. The performance of the models was evaluated using two different types of tests, as detailed in the subsequent subsections. In the first test, quantitative metrics of the models were measured to determine the most suitable model for classification. In the second test, the model selected in the previous step was visualized internally, with an analysis of the feature maps. This analysis helps in identifying the features and learning layers crucial for accurate classification of current vs. angular position signals.

### 3.1. Results of Classification Metrics

In this proof of concept, we propose introducing the test set images to the six models, followed by obtaining prediction labels for each class to facilitate a comparison of the classification effectiveness. A separate test set reserved prior to the commencement of model training is utilized. The rationale for using this distinct dataset is that the models have not encountered these images during the training process, making it a suitable choice for validation of the proof of concept. The test set consists of a collection of graphs depicting various combinations of current versus angular position, including 19 images of a healthy motor, 18 images of a motor with one broken bar, 247 images of a motor with two broken bars, 1076 images of a motor with three broken bars, 93 images of a motor with four broken bars, 361 images of a motor with five broken bars, and 1302 images of a motor with six broken bars.

Several quantitative measures were considered to assess the classification results of the CNNs examined in this study. The metrics most frequently employed include accuracy, precision, recall, and F1 score. Accuracy (Equation (4)) gauges the overall correctness of the classification by dividing the number of correctly classified instances (true positives and true negatives) by the total number of instances. Precision (Equation (5)) evaluates the proportion of true-positive predictions among all positive predictions, reflecting the model’s capability of minimizing false positives. Recall (Equation (6)), often termed sensitivity, measures the proportion of true-positive predictions among all actual positive instances, capturing the model’s capability of reducing false negatives. The F1 score (Equation (7)) amalgamates precision and recall, offering a singular value symbolizing the equilibrium between the two metrics.

(4) $$ACC=\frac{\left(TP+TN\right)}{TP+TN+FP+FN}$$

(5) $$Precision=\frac{TP}{TP+FP}$$

(6) $$Recall=\frac{TP}{TP+FN}$$

(7) $$F1-score=2\;\cdot \;\frac{Precision\;\cdot \;Recall}{Precision+Recall}$$

where *TP* represents true positives, *FP* denotes false positives, *TN* signifies true negatives, and *FN* represents false negatives.

These metrics were calculated from a confusion matrix for each model. The confusion matrix [56] is a matrix representation in which columns indicate the predictions for each class, and rows denote the actual instances per class. The matrix is presented in terms of percentage values. Table 3 displays the confusion matrices for each architecture, while Table 4 reveals the percentage of global metrics for each architecture used in the test suite. Additionally, Table 4 features the P. Time column, signifying the processing time necessary to handle the entire test set for each architecture (in seconds). To ensure precise measurements, initial and final timestamps were recorded when channeling the complete set of tests through each CNN, ensuring consistent conditions throughout the evaluation process, including the same starting parameters like temperature, system configuration, and other pertinent variables.

All architectures demonstrated high performance in the classification of the test set, indicating their effectiveness in detecting broken bars in induction motors. However, the training losses, accuracy, and training time are important factors to consider, as they can provide insights into the model complexity and efficiency, as addressed in Section 2.3. Among the considered architectures (Inception V4, NasNETMobile, ResNET152, SeNET154, and VGG19), VGG19 emerged as the selected architecture for this task. With accuracy, precision, recall, and F1 score of 0.998, 0.994, 0.994, and 0.994, respectively, VGG19 showcased the ability to accurately classify different types of broken bars.

### 3.2. Internal Analysis of the Fault Detection System

**Figure 5:**
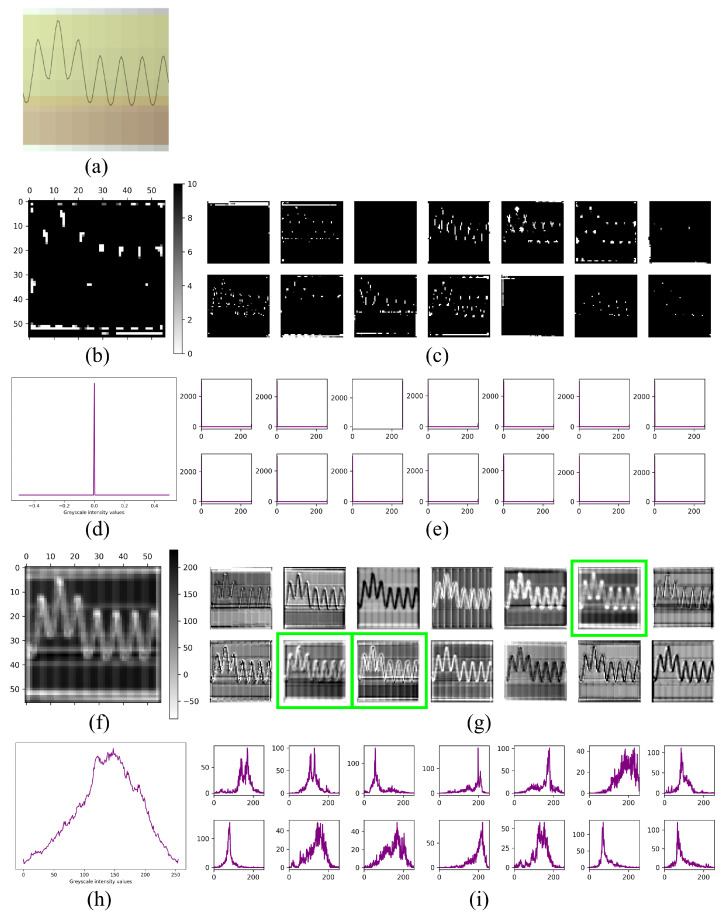
VGG19 feature maps: (**a**) Input image of a broken bar. (**b**) Feature map 72 from block 3, layer 1 of the untrained CNN. (**c**) Sample feature maps from the same block and layer of the untrained CNN. (**d**) Average grayscale histogram of filters from the untrained block and layer. (**e**) Histograms of individual filters from the untrained block and layer. (**f**) Feature map 72 from block 3, layer 1 of the trained CNN. (**g**) Sample feature maps from the trained block and layer, with significant maps highlighted in green. (**h**) Average grayscale histogram of filters from the trained block and layer. (**i**) Histograms of individual filters from the trained block and layer.

In this subsection, we delve into an analysis of the internal dynamics of the fault detection system by scrutinizing the feature maps of the VGG19 network, contrasting its behavior before and after domain-specific training. To accomplish this, we passed an image processed using our proposed preprocessing technique through the network (Figure 5a). ImageNet [57] is a vast database widely used in the deep learning community, consisting of millions of labeled images spanning numerous categories. Its sheer size and diversity have made it a foundational tool for the pretraining of convolutional neural networks (CNNs). When a CNN such as VGG19 is pretrained on ImageNet, it assimilates generic features relevant across a wide spectrum of visual data. This forms a rich basis, which can later be fine-tuned to specialize in specific tasks.

Given this context, our attention is specifically directed towards feature map 72 from convolutional block 3, layer 1. In the VGG19 model pretrained solely on ImageNet, this feature map appears dormant, as evidenced by the mostly black representation in the image (Figure 5b). This could indicate that the generic features learned from ImageNet are not immediately relevant or activated for our specific task. However, after our targeted training, there was a discernible surge in activation across the feature maps. This is particularly prominent in the aforementioned layer, suggesting that our training endowed the network with specialized knowledge, making it more sensitive to features of interest in our task (Figure 5f).

A key observation is the discernment of the gradient color grid in the image background, which is proposed as a preprocessing step. This grid pattern was effectively detected by the network filters, facilitating the subdivision of the waveform of the signals at 10-degree intervals. By leveraging this grid pattern, activation of the feature maps in specific regions that delineate the signal behavior becomes apparent. To demonstrate this behavior, examples of activated feature maps within this layer using the pretrained model (Figure 5c) and post training for current signal classification (Figure 5g) are provided. Feature maps that display higher histogram significance are outlined within green-colored rectangles.

The grayscale histograms associated with this layer were analyzed to further understand the behavior of the feature maps. Before training, the histograms primarily lean to the left, hinting at minimal activation within the feature map (Figure 5e). In contrast, post training, there was a discernible shift in the histograms towards a central tendency, accompanied by a notable presence of values on the right (Figure 5i). Such a shift implies pronounced activation within this layer when identifying current signals.

To provide a comprehensive visualization of the findings, an average plot of the grayscale histograms before (Figure 5d) and after (Figure 5h) network training is presented. The initial histogram plot indicates a full trend on the left, suggesting minimal activation within the pretrained CNN. Conversely, the average histogram after training displays a distribution that trends towards normality, with a slight bias to the right. This observation implies an increasingly active response from the feature maps within these images, as visually represented by higher intensities of white. Such visual representations facilitate the identification of the most informative convolutional layer within the model, paving the way for a deeper understanding of the internal analysis of the fault detection system.

## 4. Discussion

In this study, convolutional neural networks (CNNs) were utilized to develop a system capable of identifying up to seven potential diagnostic scenarios in induction machines. The system used images of current signals plotted against their angular positions as input. Uniquely, these images, showcased on a color gradient background, were obtained from the FEMM software. This research provides a comparative analysis of various CNN architectures with the aim of identifying the most effective model and the activation layer that significantly influences the learning process. This contribution to the field of induction machine fault detection can potentially enhance the performance and accuracy of diagnostic systems. The proposed system was specifically designed to recognize signals presented in a two-dimensional graph. The conducted comprehensive evaluation demonstrates the optimal performance of the selected architectures for this application. Furthermore, the evaluation of feature maps confirms that the additional preprocessing technique outlined in this article effectively aids in the identification of current signal behavior by the model, thereby enhancing deep learning capabilities.

The subsequent sections of this discussion address the following aspects: analysis of results, structural analysis of the chosen model, utilization of computational resources, and limitations of this work with respect to fault detection systems.

### 4.1. Analysis of Results

The implementation of the proposed preprocessing step led to a significant enhancement of the overall performance of the classification system, as depicted in Table 3. This improvement was consistently observed across all network architectures, indicating the effectiveness of the preprocessing technique. The impact of the degraded background can be further visualized in Figure 3, which illustrates the evolution of losses before and after the application of the degraded background. It becomes evident that the introduction of the degraded background contributed to lower losses during the initial training epochs for all models. This trend suggests that the inclusion of the degraded background facilitated a more efficient learning process for the system.

Upon analysis of the learning process of the different architectures, distinct dynamics emerged. Specifically, the VGG19 architecture displayed the highest initial losses among all the models. However, it showed a decay slope throughout the training, indicating its capacity to learn time-series patterns. Conversely, the evolution of training accuracy (Figure 4) revealed that VGG19 initially has the lowest learning curve among all models. However, starting from epoch 12, VGG19 exhibited a sharper increase in the slope of accuracy, ultimately achieving a notably high accuracy value.

In the classification results from the VGG19 model, an interdependence manifested between classes B4, B5, and B6, as quantified in the confusion matrix (Table 3). This behavior exemplifies the intrinsic overlaps present within feature spaces of these closely associated classes, highlighting the proficiency of CNN architectures in discerning subtle fault severity gradations. The 1–2% divergence observed in the confusion matrix underscores the intricacies of complex system diagnostics, where attaining absolute statistical separability between analogous classes is unlikely. Furthermore, it underscores the integral role of seasoned field experts in augmenting the precision of model-driven diagnostics, contributing their wealth of empirical insights to the analytical process.

Considering the observations of the training evolution, the VGG19 architecture was selected as the most suitable model for the system. This decision was supported by the proposed preprocessing technique, which allowed the CNNs to more effectively discern the features associated with failures in the induction machine images. Taking these factors into consideration, it was determined that the chosen model demonstrated superior performance, effectively meeting the objectives of the study.

### 4.2. Structural Analysis of the Model

In an effort to provide a more comprehensive understanding, an analysis not frequently seen in the technical literature was carried out to examine the impact of the training process on the activation of the model. To achieve this, a comparison was performed between the trained model and the same model without training, using default ImageNet weights. The feature maps illustrated in Figure 5g correspond to those visualized from the trained model, while Figure 5c displays the feature maps from the default model. Characteristics ranging from most to least representative are indicated by a shift from white to black, respectively. Strikingly, the visualizations from the default model showed a dominant presence of the color black, signaling a lack of representative characteristics in the model. In contrast, the trained model exhibited significant activation representation. Furthermore, grayscale histogram distributions were employed to visualize these maps for both the default model (Figure 5e) and the trained model (Figure 5i). The histograms exhibited a central–right trend when the model recognized the trained feature map, in contrast to a leftward trend when the model employed default weights.

Furthermore, in this layer, the presence of a gradient background in the form of a grid played a significant role. Figure 5f showcases the activation of regions corresponding to the grid and its neighboring pixels, as influenced by the signal waveform in feature map 72 from convolutional block 3, layer 1. In contrast, when the model employed default training weights (Figure 5b), no activation was observed in the same feature map. The structural analysis of the VGG19 model highlighted its efficacy in capturing intricate patterns and features, leading to successful classification of broken bars in induction motors. Additionally, the exploration of feature activations within the model provided insights into the specific layer where learning capacity was most pronounced.

### 4.3. Computational Resources

CNN architectures require significant computational processing power, which is why the models investigated in this study were trained using a cloud service. Cloud services are remote computing platforms that offer virtual machines and storage, allowing users to access robust computing capabilities without the need for dedicated hardware. In this study, we utilized the Amazon EC2 service, which provided NVIDIA T4 GPUs (graphics processing units), which are well-suited for tasks requiring Nvidia^®^ CUDA. The models were developed using the fastai library in PyTorch. The training process used fastaiV2, while the feature maps were analyzed directly using PyTorch due to its flexibility in manipulation of neural networks. The models are compatible with both libraries, as fastai is built on top of PyTorch.

To ensure a fair comparison among all architectures, a batch size of eight images was established. This stipulation means that each network passed batches of eight images until processing all the images in the training set were processed, signifying the completion of a training epoch. In evaluating the training time per epoch, as illustrated in Figure 4, it was observed that architectures with greater depth, such as SeNET154, exhibited similar processing times to that of the shallower architecture of VGG16. Both architectures took longer per epoch, primarily due to the batch size. Training these models necessitates specific tools and comes with associated implementation costs. Choosing the appropriate architecture for implementation requires careful consideration. However, once the models have been trained, there are available low-cost deep learning embedded cards that have shown efficiency in implementing models trained with the VGG19 architecture. As a result, implementation of the trained models is deemed feasible. The importance of comparing different architectures to glean insights into the necessary resources, training time, precision, and procedures needed for future research in this domain is emphasized.

### 4.4. Limitations and Future Work

This work is subject to limitations that present opportunities for future research and development. We directed our efforts towards the comparison of different architectures, aiming to identify the optimal architecture for a failure detection system. Using signals from a 28-bar induction motor simulated in FEMM provided a solid foundation, setting the stage for further research to extend this methodology to induction motors with diverse numbers of bars. It is crucial to recognize that current signatures vary based on the individual characteristics of each motor, including winding configurations, bus counts, rotor design, and other structural attributes. Given this understanding, the core methodology of analyzing current signals, in principle, is adaptable to the diagnosis of motors with diverse bar counts and winding schemes. However, this would necessitate extensive simulations tailored to different motor archetypes to ensure accurate diagnostics. Simulating failures under controlled conditions across varied engine designs allows this data-centric methodology to be fine-tuned, broadening its applicability to precise failure detection and diagnosis in a multitude of industrial settings. We envisage future studies including a wider spectrum of motors, like those with 22, 24, 26, 28, 30, and 32 bars, offering a comprehensive evaluation.

While it is true that a rotor may need replacement regardless of the number of broken bars, accurately diagnosing the degree of damage has several strategic advantages. Precise knowledge of the damage offers invaluable insights into wear patterns, subsequently informing predictive maintenance models. This clarity promotes effective maintenance scheduling, mitigating operational interruptions. Such information is paramount in anticipating potential machinery failures and enacting timely interventions. Furthermore, it serves as a platform for implementing temporary operational adaptations, aiming to prolong the equipment’s life until scheduled maintenance or replacement. By harnessing the power of automation with CNNs, we underscore the efficacy and adaptability of this diagnostic process across various machinery contexts.

Our research covered seven potential scenarios of broken bars, but the real promise lies in broadening the scope. Delving into the classification application of the system for diverse failure types, especially where the current is an indicative factor, can birth a more adaptable and resilient diagnostic tool.

We recognize that training and comparing multiple architectures demands significant computational resources. But this challenge also hints at an exciting opportunity: the pursuit of efficient computation. The realm of deep learning often leans on high-performance computing systems or GPUs, but we foresee a future brimming with optimized algorithms, model compression, and computation-saving techniques that can further refine our approach.

Deep learning’s dynamic nature means the field is always on the move, making it thrilling to be part of. While certain architectures and methods might evolve, our work resonates with the growing importance of AI in industrial contexts. We are optimistic that as deep learning advances, so will the prowess of fault detection systems. Our research, while shedding light on certain areas of enhancement, predominantly underscores the myriad avenues waiting to be explored. By diving deeper into these domains, we can continually refine our approach, staying in tandem with the latest in the field.

## 5. Conclusions

This research contributes to the field of fault detection in induction motors by demonstrating the efficacy of convolutional neural networks (CNNs) in accurately identifying and quantifying broken bar faults in squirrel-cage induction motors. The application of CNNs to the analysis of current and angular position data results in precise detection of broken bars, achieving an accuracy of 99%. A comprehensive assessment of six distinct CNN architectures (Inception V4, NasNETMobile, ResNET152, SeNET154, VGG16, and VGG19) underscores VGG19 as the most optimal model for this specific classification task.

The integration of a novel preprocessing technique involving a gradient-colored grid added to image backgrounds significantly enhances the CNN classification performance. Structural analysis of the selected model, VGG19, reveals the activation of specific feature maps, underscoring the crucial role of the degraded background grid pattern in facilitating the recognition of current signal behavior.

The proposed system presents tangible benefits, particularly in preventive and corrective maintenance strategies. By enabling early failure diagnosis within a short time frame, this system has the potential to reduce operational disruptions and extend the operational life of induction motors. This research marks a significant step forward in fault detection for induction motors, offering a robust approach and highlighting its practical applicability and far-reaching advantages.

## Figures and Tables

**Table 1:** Summary of the proposed approach for diagnosis of broken rotor bars in induction machines.

Goal	Develop and evaluate a CNN-based system for automatic detection and quantification of broken rotor bars in induction machines using current signal vs. angular position images.
Methods	Current vs. angular position images were obtained from FEMM simulation of a 28-bar induction machine. A novel image preprocessing technique using a gradient-colored background grid was applied. Six CNN architectures (Inception V4, NasNETMobile, ResNet152, SeNET154, VGG16, and VGG19) were implemented and compared. Models were trained by tuning hyperparameters like batch size, learning rate, optimizers, regularization, etc. Models were evaluated using metrics like accuracy, precision, recall, and F1 score. Feature maps of the best model, VGG19, were analyzed before and after training.
Results	The VGG19 model achieved the highest accuracy of 99% in classifying broken bars. Image preprocessing enhanced model performance significantly. Training activated specific feature maps related to current signals. Analyzing feature maps provided insights into how the model learns.
Advantages	The system enables automated broken bar detection and quantifies the degree of damage. It achieves high accuracy comparable to that of traditional methods, allowing for rapid diagnosis to reduce downtime. The approach is widely applicable for varying induction machine designs.
Limitations	The system is currently limited to a 28-bar induction machine. Considerable computational resources are needed to train the models. The dataset is obtained through simulation. This research focuses solely on detecting broken bar faults.

**Table 2:** Samples for training of the automatic broken bar detection system using a CNN. Samples refer to the signals of broken bars obtained for detection. Combinations represent the variation in the initial angle, rotating through 90 degrees. Train, Val, and Test indicate the sets of images used for training, validation, and testing of the CNN models, respectively. Data Augmentation represents the number of final images after applying data augmentation and data balancing techniques.

	Broken Bar Database							
	Current Signals vs. Angular Position					Data Augmentation		
Broken Bars	Samples	Combinations ^*^	Train	Val	Test	Train	Val	Test
Healthy	1	90	63	8	19	2558	442	19
One	1	90	61	11	18	2536	464	18
Two	14	1260	844	139	247	2578	422	247
Three	65	5850	3685	649	1076	2572	428	1076
Four	410	410	269	48	93	2542	458	93
Five	1764	1764	1216	187	361	2536	464	361
Six	6586	6586	4456	828	1302	2528	472	1302

${}^{*}$ Number of broken bars and their relative positions.

**Table 3:** Comparison of confusion matrices in percentage for six CNN architectures: Inception V4 (1), NasNETMobile (2), ResNET152 (3), SeNET154 (4), VGG16 (5), and VGG19 (6). Classifications include a healthy machine (H) and machines with one to six broken bars (B1–B6).

	(1)	Inception V4								(2)	NasNETMobile						
		H	B1	B2	B3	B4	B5	B6			H	B1	B2	B3	B4	B5	B6
True Class	H	**100**	0	0	0	0	0	0		H	**100**	0	0	0	0	0	0
	B1	0	**100**	0	0	0	0	0		B1	0	**100**	0	0	0	0	0
	B2	0	0	**100**	0	0	0	0		B2	0	0	**100**	0	0	0	0
	B3	0	0	0	**100**	0	0	0		B3	0	0	0	**100**	0	0	0
	B4	0	0	0	0	**97**	3	0		B4	0	0	0	0	**97**	3	0
	B5	0	0	0	0	1	**97**	2		B5	0	0	0	0	1	**97**	2
	B6	0	0	0	0	0	2	**98**		B6	0	0	0	0	0	2	**98**
	(3)	ResNET152								(4)	SeNET154						
		H	B1	B2	B3	B4	B5	B6			H	B1	B2	B3	B4	B5	B6
True Class	H	**100**	0	0	0	0	0	0		H	**100**	0	0	0	0	0	0
	B1	0	**100**	0	0	0	0	0		B1	0	**100**	0	0	0	0	0
	B2	0	0	**100**	0	0	0	0		B2	0	0	**100**	0	0	0	0
	B3	0	0	0	**100**	0	0	0		B3	0	0	0	**100**	0	0	0
	B4	0	0	0	0	**97**	3	0		B4	0	0	0	0	**100**	0	0
	B5	0	0	0	0	1	**97**	2		B5	0	0	0	0	0	**98**	2
	B6	0	0	0	0	0	2	**98**		B6	0	0	0	0	0	2	**98**
	(5)	VGG16								(6)	VGG19						
		H	B1	B2	B3	B4	B5	B6			H	B1	B2	B3	B4	B5	B6
True Class	H	**100**	0	0	0	0	0	0		H	**100**	0	0	0	0	0	0
	B1	0	**100**	0	0	0	0	0		B1	0	**100**	0	0	0	0	0
	B2	0	0	**100**	0	0	0	0		B2	0	0	**100**	0	0	0	0
	B3	0	0	0	**100**	0	0	0		B3	0	0	0	**100**	0	0	0
	B4	0	0	0	0	**98**	2	0		B4	0	0	0	0	**99**	1	0
	B5	0	0	0	0	0	**99**	1		B5	0	0	0	0	0	**99**	1
	B6	0	0	0	0	0	1	**99**		B6	0	0	0	0	0	2	**98**
		Predicted Class									Predicted Class						

**Table 4:** Performance metrics for different CNN architectures included in this study.

Architecture	Accuracy	Precision	Recall	F1-Score	P. Time
Inception V4	0.997	0.989	0.989	0.989	6.081
NasNETMobile	0.998	0.994	0.994	0.994	6.187
ResNET152	0.997	0.990	0.990	0.990	6.276
SeNET154	0.998	0.994	0.994	0.994	7.874
VGG16	0.998	0.994	0.994	0.994	6.143
VGG19	0.998	0.994	0.994	0.994	6.123

## Data Availability

Data sharing is not applicable to this article.
